# Recognition of Targets in SAR Images Based on a WVV Feature Using a Subset of Scattering Centers

**DOI:** 10.3390/s22218528

**Published:** 2022-11-05

**Authors:** Sumi Lee, Sang-Wan Kim

**Affiliations:** 1 Department of Geoinformation Engineering, Sejong University, 209 Neungdong-ro, Gwangjin-gu, Seoul 05006, Korea; 2 Department of Energy Resources and Geosystems Engineering, Sejong University, 209 Neungdong-ro, Gwangjin-gu, Seoul 05006, Korea

**Keywords:** synthetic aperture radar, scattering center, world view vector, recognition, SAMPLE

## Abstract

This paper proposes a robust method for feature-based matching with potential for application to synthetic aperture radar (SAR) automatic target recognition (ATR). The scarcity of measured SAR data available for training classification algorithms leads to the replacement of such data with synthetic data. As attributed scattering centers (ASCs) extracted from the SAR image reflect the electromagnetic phenomenon of the SAR target, this is effective for classifying targets when purely synthetic SAR images are used as the template. In the classification stage, following preparation of the extracted template ASC dataset, some of the template ASCs were subsampled by the amplitude and the neighbor matching algorithm to focus on the related points of the test ASCs. Then, the subset of ASCs were reconstructed to the world view vector feature set, considering the point similarity and structure similarity simultaneously. Finally, the matching scores between the two sets were calculated using weighted bipartite graph matching and then combined with several weights for overall similarity. Experiments on synthetic and measured paired labeled experiment datasets, which are publicly available, were conducted to verify the effectiveness and robustness of the proposed method. The proposed method can be used in practical SAR ATR systems trained using simulated images.

## 1. Introduction

Over the last few years, automatic target recognition using synthetic aperture radar (SAR ATR) has increasingly become important as a crucial means of surveillance [1,2,3,4]. SAR images can be obtained in most weather types, day and night, and at a high resolution [5,6,7,8]. Based on these characteristics, SAR ATR algorithms have been evaluated on several targets, including ground-based vehicles, aircrafts, and vessels, which are challenging for military operations [9]. However, the sensitivity of SAR to sensor parameters, target configurations, and environmental conditions make it challenging to implement SAR ATR [10,11,12].

SAR ATR algorithms can be divided into three basic steps: detection, discrimination, and classification [13,14]. The first two steps are intended to extract potential target areas and remove false alarms [15]. The purpose of target classification is to automatically classify each input target image obtained by target detection and discrimination [16]. A large number of efforts have been made to achieve robust SAR ATR. However, the classification performances under extended operating conditions (EOCs) are insufficient for practical applications. In real-world cases, most targets are likely to be camouflaged or blocked by surrounding obstacles [11,17]. To improve the performance under EOCs within a SAR ATR system, this paper focused on the classification stage.

Target classification methods are mainly divided into model-based and feature-based methods [5]. Feature-based methods involve pattern recognition and rely solely on features to represent the target, with many SAR target templates stored in advance. Once the features of the input target are extracted, the test target is classified as the category of the matched template.

One of the promising features, attributed scattering center (ASC), provides a physically relevant description of the target [4,18,19,20,21]. According to electromagnetic theory, the high frequency scattering response of the target can be approximated by a sum of responses from multiple ASCs [22]. The classification method based on ASCs reflects the specific scattering structure of the target. In Refs. [16,23], an ASC-based model was proposed for target classification, and they achieved higher accuracy than when not using the ASC model. It has also been proven that the local descriptions contained in ASCs are beneficial for reasoning under EOCs [19,24]. However, there are some problems associated with point-pattern matching between test ASCs and template ASCs. Two ASC sets, even in the same class, can have different numbers of points and subtly different positions according to the SAR sensor operating conditions [25]. Therefore, one-to-one correspondence is impractical, and similarity measurements of the two point sets are highly complex.

To solve these problems, several researchers have attempted to design optimal ASC-based methods. In Ref. [26], the researchers introduced the world view vector (WVV) to reconstruct the ASC features, then the weighted bipartite graph model (WBGM) was used. As the WVV provides a robust description of the point location and spatial relationship between the two sets, each weight on the line was allocated by computing the WVV-based similarity of the two ASC sets. Therefore, the direct one-to-one matching problem was solved. In Ref. [24], a similarity evaluation method for two ASC sets was introduced by exploiting the structural information contained in the ASC set. Once ASC matching was conducted using the Hungarian algorithm, point similarity and structure similarity were fused to evaluate the overall similarity of the two ASC sets based on the Dempster–Shafer theory of evidence. Meanwhile, a target reconstruction based on ASC was employed to avoid precise one-to-one correspondence and complex similarity measures [19,27]. Using the neighbor matching algorithm, only the template ASCs related to the test ASCs were selected to reconstruct the template image, whereas all the test ASCs were used to reconstruct the test image. Then, image correlation was employed to effectively measure the similarity between the two ASC sets.

As a dataset for experiments, moving and stationary target acquisition and recognition (MSTAR), which consists of a collection of one-foot-resolution SAR images, has been widely used in the past two decades [5,9,22,28,29,30]. Unfortunately, the amount of measured data needed to build a SAR ATR system is insufficient because of practical limitations in operating sensors and targets [31]. Furthermore, measured data typically represent limited environmental conditions, with very few articulation, configuration, and clutter changes, along with few sensor collection geometry variations [8,32]. In this regard, computer-generated synthetic data could serve as a valuable tool in the development of SAR ATR systems, leading to training and testing using only synthetic data and measured data, respectively [31,33]. Synthetic data are created using electromagnetic prediction software involving a computer-aided design (CAD) model of the MSTAR. By replacing measured data with synthetic data, many EOCs can be considered close to representing the real world, which is necessary to enhance classification performance.

**Figure 1:**
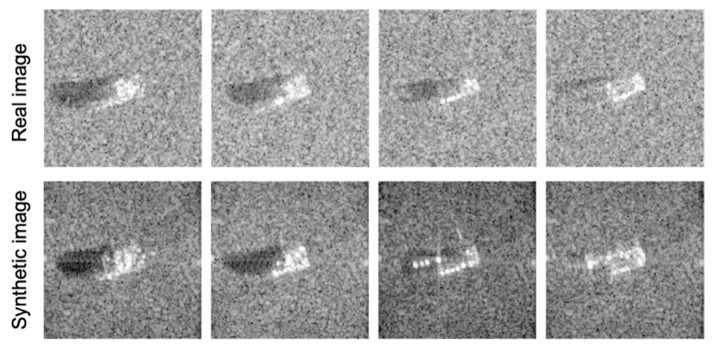
Examples of real and synthetic SAR images in SAMPLE, demonstrating substantial target differences located in the middle of the images.

However, an apparent distribution gap between synthetic and measured distributions exists due to differences in the CAD model and the real model, or the type of simulator used. Despite effort to decrease the gap [33], some parts of the target that did not appear in the measured data tend to be visible in the corresponding synthetic data (Figure 1). This could be regarded as a target occlusion situation if we only considered the target appearance of the image, which commonly happens in the real world [17,34]. Target occlusion inevitably occurs due to radar sensor operation and the external environment, such as artificial or natural objects, which make it difficult to classify the target using the traditional feature-based method. Comprehensively, a similarity measurement method where the unique features of the target can be captured is highly recommended for robustness to target occlusion in practical SAR ATR.

The previously proposed WVV-based method has a robust ATR capability using all extracted ASCs [35]. The WVV is insensitive to translation, rescaling, random perturbation, and random addition and deletion of points. However, the method is sensitive to partial differences that may arise due to simulation limitations. Further, local feature matching under imbalance from two sets is still a challenging and important point for classification problems [36,37]. It is necessary to design a classification method that is less sensitive to the partial difference between real and synthetic images. Therefore, we propose an improved WVV-based ATR method using a subset of ASCs instead of using all ASCs to focus on local similarity.

## 2. Target Classification with a WVV-Based Feature Set

**Figure 2:**
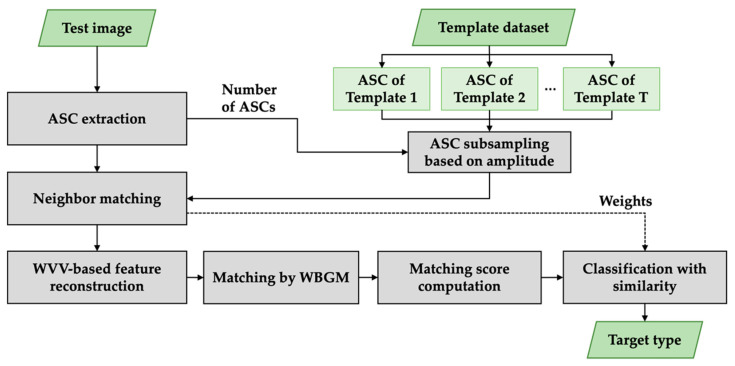
Flowchart of the proposed method.

To classify the target in SAR images, we designed the classification algorithm in terms of local features matching. The ASCs were used as the unique features of the target in the SAR image. Figure 2 illustrates a flowchart of the proposed method. The flowchart is divided into five main steps: extraction of the scattering centers, sub-sampling of scatter centers based on amplitude, neighbor matching, WVV-based feature reconstruction, and similarity measurement. The ASCs of the template dataset extracted from each template image were prepared offline in advance. The value of T in Figure 2 is the number of template samples. In the classification algorithm, the extraction of scattering center from a test image was first employed. To analyze the local similarity and the imbalance problem in two ASC sets, some of the template ASCs were then selected by amplitude-based subsampling, where the number of test ASCs was exploited to adjusting the number of template ASCs. Afterwards, neighbor matching was applied to partially concentrate on the related points between the test ASCs and template ASCs. The subset of ASCs was used to reconstruct the world view vector feature set, thus considering the point similarity and structure similarity simultaneously. Later, the matching scores between the two ASC sets were calculated using weighted bipartite graph matching and then combined with several weights for overall similarity. Regarding the WVV-based similarity as the weight of bipartite graph matching, we found the optimal matching between the two sets. Finally, the overall similarity was determined to recognize the target by combining the matching score with several weights related to the matched/unmatched number of ASCs and selected/unselected number of ASCs, which could not be exclusively applied to the WVV-based similarity only. By repeating the process T times, the test image was consequently classified.

### 2.1. Extraction of Scattering Centers

Several algorithms are available for extracting scatter centers in the image domain [38,39]. CLEAN is one of the most used algorithms for extracting scatter centers from SAR images [16,17,19,24,26,27,40,41,42,43]. The CLEAN algorithm employs a filter derived from the point spread function (PSF), which is given by:

(1) $$\mathrm{PSF}\left(x,y\right)=\left(e^{j\frac{4\pi f_{c}}{c}\left(x+\theta_{c}y\right)}\frac{4f_{c}B\Omega }{c^{2}}\right)\sin c\left(\frac{2B}{c}x\right)\sin c\left(\frac{2f_{c}\Omega }{c}y\right)$$

where $\left(x,y\right)$ denotes the position of the scattering center; c is the speed of light; $f_{c}$ is the center frequency; $\theta_{c}$ is the center azimuth; B is the frequency bandwidth of the radar; and Ω is the azimuth aperture. To extract the scattering centers properly, the filter used in the CLEAN algorithm has to incorporate the same smoothing window $\omega \left(x,y\right)$ used during image formation, resulting in:

(2) $$h\left(x,y\right)=\mathrm{psf}\left(x,y\right)\omega \left(x,y\right)$$

This work used a −35 dB Taylor Window that was employed by the SAMPLE dataset [31].

The CLEAN algorithm searches for the highest-amplitude pixel in the SAR image and records its amplitude $A_{i}$ and its image coordinates ($x_{i}$, $y_{i}$). Then, the filter $h\left(x,y\right)$ shifts to the center of the pixel location and is multiplied by $A_{i}$. Assuming a point spread function (PSF) with a corresponding amplitude, the response of the imaging system is removed from the complex image [44]. In general, the iterative process is repeated with the residual image until a predetermined number of ASCs are extracted, or the amplitude of the extracted scattering point is less than the threshold value.

**Figure 3:**
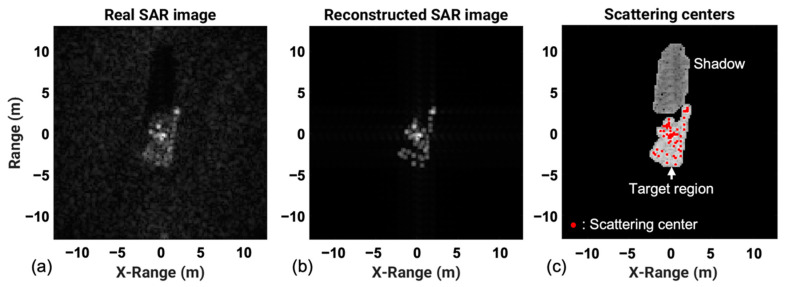
Examples of scattering centers extracted using CLEAN: (**a**) SAR image, (**b**) reconstructed SAR image, and (**c**) extracted scattering centers with the segmentation results.

In this study, we extracted scattering centers located in a target region if its amplitude was greater than or equal to a threshold, which was intended to limit the number of ASCs. The target region and shadow of a SAR image can be separated by some conventional segmentation algorithms, including the K-means, the Otsu’s method, and the iterated conditional modes (ICM). In this experiment, the ICM in Ref. [45] was used, but the ICM processing speed was improved by using the initial segmented image by the K-means, instead of the SAR image, as the input data of the ICM. Figure 3 shows the results of our scattering-center extraction task using CLEAN. The reconstructed SAR image, using a total of N scattering centers, shown in Figure 3b, was very similar to the real SAR image. The segmentation results and the extracted scattering centers located only inside the target region are shown in Figure 3c. At the end of the extraction process, the scattering centers were stored in an N × 3 matrix, $\left\{A_{i},x_{i},y_{i}\;|\;i=1,\;2,\ldots ,\;N\right\}$. For the extracted scattering centers, the coordinates of the range (in other words, the slant range) were converted to the ground range to facilitate the matching analysis of scattering centers between SAR images taken from different depression angles, as follows:

(3) $$x_{i}^{g}=x_{i}/cos\left(\theta_{depression}\right)$$

where $\theta_{depression}$ denotes the depression angle. Hereafter, $x_{i}$ is referred to as ground range coordinated, $x_{i}^{g}$.

In this paper, we used the ASC location ($x_{i},y_{i}$) and normalized amplitude ($\left|A_{i}^{Norm}\right|)$ for the SAR ATR. The locations describe the spatial distribution of ASCs, and the normalized amplitudes reflect the relative intensities of different ASCs. Therefore, the direct relevance to the target physical structures benefits the ATR performance. The *N*-scattering center feature set G was defined as follows [26]:

(4) $$G=\{\left(x_{i},y_{i},\;\left|A_{i}^{\mathrm{Norm}}\right|\right)\;|\;i=1,2,\ldots ,N\}$$

### 2.2. Amplitude-Based Selection

The apparent distribution difference between the amplitude distributions of synthetic and real SAR images is a challenge in training synthetic data. The statistical differences between the synthetic and measured data of SAMPLE were investigated by Ref. [33] to plot histograms of the image means and variances. The synthetic data tended to have a lower mean and variance than the measured data. The mean image difference was approximately 0.1 when we extracted scattering centers from real and synthetic images. There was a difference in the number of SCs extracted between the synthetic and real images. This could be due to the clear differences in the overall structure of the targets, as well as fine differences in the target details. However, it could also be due to the use of unequal minimum amplitudes to alleviate the difference in amplitude values. Before neighbor matching, a subset of the scatter points in the template image was selected in the order of their amplitude values.

The number of scatter points in subset $M^{\prime }$ was equal to the number of scatter points in the test image. However, the amplitude of the scattering points extracted from SAR images varied depending on subtle changes in the target’s pose and imaging geometry (e.g., depression angle), and it is desirable to extract the points with a small buffer according to $A_{ratio}$ as follows:

(5) $$M\prime =\;\min(M,\;N\times A_{\mathrm{ratio}})$$

where *N* and *M* are the numbers of scatter points in the test and template images, respectively. More ASCs in the template (synthetic) image can be expected (i.e., $A_{ratio}$ > 1), considering the possible partial and random occlusion of the test image and the full set of scattering centers of the synthetic image (at least no occlusion). In addition, a maximum value should be set to control the imbalance of extracted ASC numbers between the real and synthetic images. In our experiments, the best results were achieved when the $A_{ratio}$ was around 1.3, which was used in the following analysis.

### 2.3. Neighbor Matching

As shown in Figure 1, scattering centers with strong amplitudes in synthetic images are often invisible in real SAR images. In addition, the scattering centers of a part of the target may not appear in the real image because of the occlusion of the target. Therefore, considering the above reasons (difference from synthetic images and target occlusion), we focused on local similarity rather than identification using all scattering centers. For local similarity, the WVV descriptor should be reconstructed using scattering centers in the target overlap area (i.e., adjacent scattering centers). In this study, after selecting only the adjacent points between two sets of scattering centers through the neighbor-matching algorithm, we evaluated the similarity. First, the positions of the test ASCs were taken as the centers to form a binary region combined by all circles. When the template ASC was in the constructed binary region, it was selected; otherwise, it was discarded. The positions of the template ASCs were then reversed. The radius set for neighbor matching was chosen to be {0.3, 0.4, and 0.5 m} based on the resolution of MSTAR images, as well as the experimental observations [19,27].

**Figure 4:**
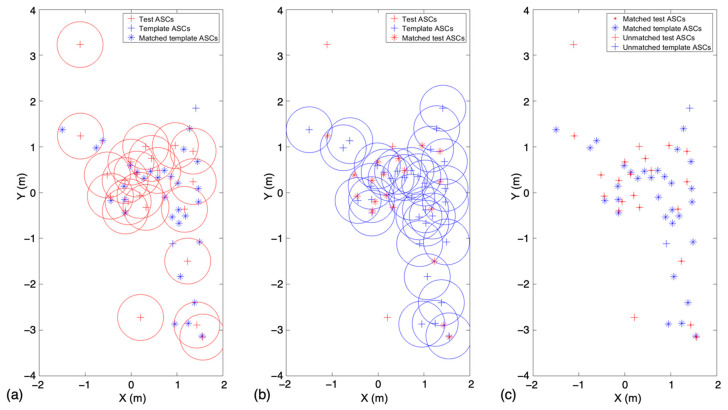
Example of neighbor matching: (**a**,**b**) neighbor matching based on the coordinates of the test ASC and template ASC, respectively. (**c**) Result of neighbor matching.

Figure 4 presents an illustration of neighbor matching when the radius was set to 0.5 m. Neighbor matching was first conducted on the test ASCs (Figure 3a) and then on the template ASCs (Figure 3b). The corresponding matching results are shown in Figure 3a,b. Matched template ASCs, unmatched template ASCs, matched test ASCs, and unmatched test ASCs are represented by different markers. As shown in Figure 3c, the ratio of unmatched to matched points can be used to distinguish different targets. It is clear that the selected number of ASCs in the template was smaller when the type was not similar. Therefore, the matching results can provide discriminability for correct target recognition.

### 2.4. WVV-Based Feature Reconstruction

To solve the difficulties of similarity calculation, as mentioned before, the correspondence was established based on the existing feature descriptor, the WVV [26]. Test ASCs and matched template ASCs were used to construct each WVV-based feature set. The detailed procedure of the WVV-based feature reconstruction is illustrated in Algorithm 1 [26]. First, WVV establishes a polar coordinate system, taking the ith scattering center as the origin. The location of i is represented by the polar radii and polar angles of the remaining SCs. Next, the $WVV_{i}$ is defined by sorting the polar radii according to their polar angles, and the radii are linearly interpolated at 1° intervals. The WVV is mapped into a vector of length 360. Finally, to avoid sensitivity to rescaling, the elements in the interpolated WVV are normalized by the maximum element. After iteration as the number of scattering centers, the scattering center feature set will consequently be the WVV-based feature set.

**Algorithm 1:** WVV-based scattering center feature reconstructionInput: Scattering center feature set $G=\{\left(x_{i},y_{i},\;\left|A_{i}^{Norm}\right|\right)|i=1,2,\ldots ,N\}$. For *i = 1*:*N* 1. Establish a polar coordinate system with the origin at ith point. 2. Compute the polar radii $r_{ik}$ and the polar angles $\theta_{ik}\;\left(k=1,\ldots ,N,\;k\neq i\right)$ of the rest N−1 points. 3. Sort the polar radii $r_{ik}$ corresponding to their polar angles $\theta_{ik}$ and define ith WVV as $WVV_{i}=\left\{r_{ik}|k=1,\ldots ,N;k\neq i;\theta_{ik}\leq \theta_{ik+1}\right\}$. 4. Interpolate linearly the polar radii. 5. Construct the interpolated *WVV*, $WVV_{i}^{interp}$ which consists of $r_{ij}\;\left(j=1,\ldots ,\;360\right)$. 6. Normalize the $r_{ij}$ by the maximum element, $r_{ij}^{Norm}=r_{ij}/\underset{k=1,\ldots ,360}{\max}\{r_{ij}\}$. $WVV_{i}^{interp}=${$r_{ij}^{Norm}$|j = 1, …, 360}. EndOutput: WVV-based feature set, $G^{\prime }=\{\left(WVV_{i}^{interp},\;\left|A_{i}^{Norm}\right|\right)\;|\;i=1,2,\ldots N\}$

**Figure 5:**
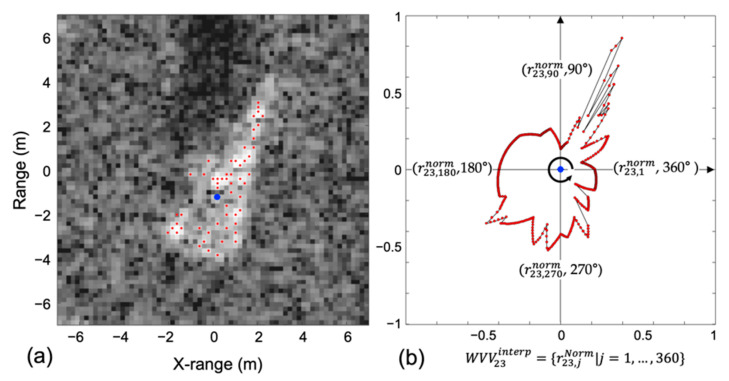
Example of WVV-based feature reconstruction using ASCs: (**a**) 56-ASC set and (**b**) interpolated 23rd WVV.

Figure 5 shows an example of WVV-based feature reconstruction using 2S1 ASCs from the SAMPLE dataset. Figure 5a,b show the 56-ASC set and interpolated 23rd WVV, respectively. In Figure 5b, the blue point at the origin is the 23rd ASC. At this point, the $WVV_{23}$ comprises the polar radii of the remaining points. Subsequently, the $WVV_{23}$ was linearly interpolated at 1° intervals, and the elements were normalized by the maximum element. After iterations, the WVV-based feature set will have 56 interpolated WVV sets and normalized amplitudes. When some ASCs are randomly removed, the WVV-based features, consisting of the remaining ASCs, can maintain their spatial structures. Therefore, the proposed method is not sensitive to random noise removal.

### 2.5. Similarity Calculation

In ATR, it is important to design a similarity measurement between the input test image and archive templates. The structural similarity between two sets of scattering centers is obtained through WVV reconstruction of the scattering centers, and several weights related to the number of scattering centers and the number of matches are proposed.

#### 2.5.1. Matching Score

After the WVV-based feature reconstruction, target classification based on point matching was conducted. The WVV provides a robust description of scattering centers. The Euclidean distance between the locations of the scattering centers is generally used when evaluating the point-to-point similarity between two sets of SCs. Meanwhile, spatial structure relationships were characterized by WVV-based features.

Comparing $G_{test}^{\prime }$, the WVV-based feature set of the test, and template dataset $\left\{B_{k}^{\prime }|k=1,\ldots T\right\}$ with Equation (4) $S(G_{test}^{\prime },B_{k}^{\prime }$) below, the most similar template was selected.

(6) $$S(G_{test}^{\prime },B_{k}^{\prime })=\sum s\left(g_{l}^{\prime },b_{l}^{\prime }\right),\;l=1,\ldots ,\min\left(M,N\right)$$

where $g_{l}^{\prime }\in G_{test}^{\prime }\;and\;\;b_{l}^{\prime }\in \;B_{k}^{\prime }$ are the matching pairs and *N* and *M* are the number of SCs in the test and *k*-th template, respectively.

There was no strict one-to-one correspondence between the two sets. We needed to find an optimal assignment to maximize *S* between the two point sets. Based on weighted bipartite graph matching (WBGM), the similarity of the matching pairs was calculated as

(7) $$s\left(g_{i}^{\prime },b_{j}^{\prime }\right)=F\left(g_{i}^{\prime },b_{j}^{\prime }\right)\left(360-∥WVV_{g_{i}^{\prime }}^{interp}-WVV_{b_{j}^{\prime }}^{interp}∥{}^{2}\right)/\left(360\right)/\left(1+D\right)$$

(8) $$F\left(g_{i}^{\prime },b_{j}^{\prime }\right)=\left\{\begin{array}{c} \;1,\;\;D\left(g_{i}^{\prime },b_{j}^{\prime }\right)\leq R\\ 0,\;\;otherwise\; \end{array}\right.$$

where $D\left(g_{i}^{\prime },b_{j}^{\prime }\right)$ are the Euclidean distances between the test ASC and template ASC. If *D* is greater than *R*, which is the distance (meter) for neighbor matching, 0 is assigned to $s\left(g_{i}^{\prime },b_{j}^{\prime }\right)$ to prevent matching between points that are too far away. Accordingly, the number of matched pairs, $K_{match}$ was applied to normalize the similarity measurement.

(9) $$S_{Norm}\left(G_{test}^{\prime },B_{k}^{\prime }\right)=\frac{S\left(G_{test}^{\prime },B_{k}^{\prime }\right)}{K_{match}}$$

#### 2.5.2. Weight Design

The previously obtained WVV-based matching score did not consider the difference in the number of scattering centers that occurs when the types of targets are different. Therefore, it was necessary to properly design the weights based on the number of matches and the number of points that were not matched, and reflect them in the overall degree of similarity.

**Figure 6:**
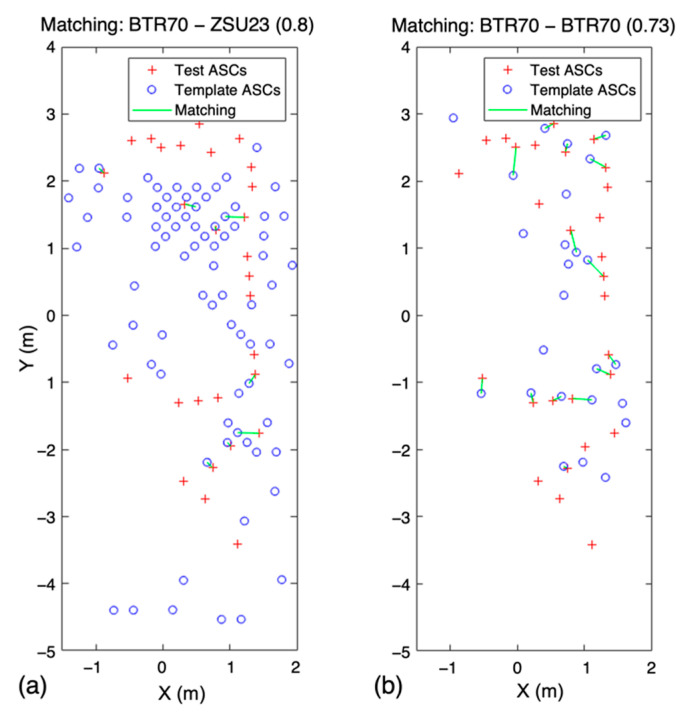
WVV-based similarity matching results between two sets of scattering centers. (**a**) Different types of test and template SCs, (**b**) same target type of test and template SCs.

Although the type was different from the test, if the template points around the test point were gathered, the WVV-based similarity was calculated to be high by neighbor matching (Figure 6a). The WVV-based matching score is higher in Figure 6a than that in Figure 6b, but in Figure 6a, the difference between the number of test and template scattering centers is substantial. Because the number of points in the template will be similar for targets of the same type, it is necessary to consider the difference in the number of points between the test and the template.

Unmatched test scattering centers (MA: Missing Alarm) and unmatched template scattering centers (FA, False Alarm) were considered in the matching score. Here, we used the following quadratic weights presented by Ref. [24]:

(10) $$w_{a}=1-{\left(\frac{s+q}{N+m}\right)}^{2}$$

where *s* and *q* represent the number of MA and FA, respectively, and *s + q* can be calculated as *N + m − 2 ×* $K_{match}$, where m is the number of matched template ASCs by the neighbor matching algorithm. Because of noise and extraction errors, it is common to observe the appearance of a few MAs and FAs. As the proportion of MAs and FAs increases, the weight $w_{a}$ decreases more quickly.

In addition, when the test and template are of the same type, the number of scattering centers within the radius tends to be large during neighbor matching. Therefore, along with $S_{Norm}$ and $w_{a}$, the ratio of the number of selected points to the total number of points was used as a weight. The overall matching score adopted in this study was defined as:

(11) $$MS=S_{Norm}(G_{test}^{\prime },B_{k}^{\prime })\;\times \;W_{a}\;\times \;n/N\;\times \;m/M$$

In Equation (11), *n/N* is the ratio of the number of scattering centers extracted from the test image to the test ASCs adjacent to the template ASCs, and *m/M* is the ratio of the number of template ASCs to the number of neighboring ASCs to the number of test ASCs.

The procedure for the proposed target WVV-based matching method is shown in Figure 2. The entire algorithm was programmed in MATLAB^®^ R2021a (9.10), employing Matlab’s Parallel Computing Toolbox. First, because it is impossible to define a one-to-one correspondence between the test and template, subsampling based on amplitude and neighbor matching was conducted to reduce the imbalance. The similarity matrix of the WVV sets was obtained using Equation (7) $s\left(g_{i}^{\prime },b_{j}^{\prime }\right)$*,* and then all the similarities were employed as weights for the WBGM. By repeating the above process based on the number of template databases, the type of test target could be determined as a category according to the template with the maximum matching score.

## 3. Experiments

### 3.1. Experimental Settings

**Figure 7:**
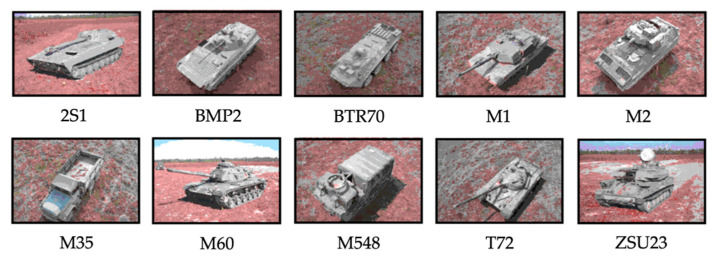
Examples of optical images of SAMPLE dataset targets.

To evaluate the classification performance of the proposed method, experiments were conducted on SAMPLE datasets under standard operation conditions (SOC) and EOC, including random and partial occlusions. The SAMPLE dataset consisted of real and synthetic SAR images using CAD models of 10-class MSTAR targets, which are listed in Table 1 [31]. Furthermore, the optical images of 10-class targets are shown in Figure 7; they are ground vehicles, carriers, and trucks (you can see more targets in SAR images and types of targets in Refs. [46,47,48,49]). The data had a spatial resolution of one foot. Unfortunately, only some parts of the SAMPLE dataset are publicly available, which is appropriate for small-scale operations. They were collected at azimuth angles of 10–80° with depression angles from 14–17°. To validate the proposed method for 10 targets, we used target chips with depression angles of 16° and 17°. In Table 1, the number of SAMPLE datasets for each target class is listed according to the depression angle.

**Figure 8:**
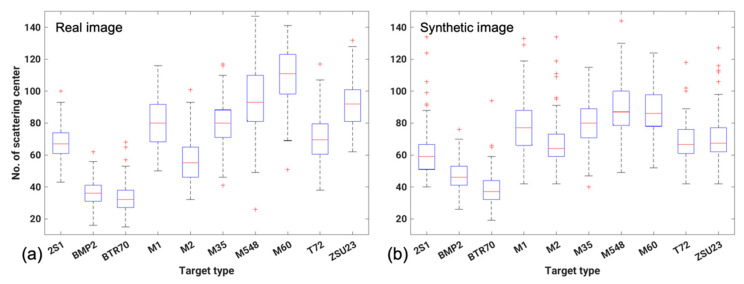
Box plot of the number of scattering centers. (**a**) Real SAR image (16°, 17°) and (**b**) synthetic SAR image (16°, 17°).

When using CLEAN, scattering centers with a minimum amplitude or higher were extracted. The distribution between the amplitude in the clutter around the target and that of the target may be considered when selecting the threshold value for the extraction of scattering centers. Because a difference in amplitude exists between real and synthetic images, scattering centers with an amplitude of 0.25 or higher for real and 0.14 or more for synthetic images were extracted considering the difference in amplitude between synthetic and real data of SAMPLE [33,50]. The statistical values of the number of extracted scattering centers are shown in Figure 8; evidently, the number of scattering centers extracted by CLEAN was different for each target. BMP2 and BTR70 had an average of 30–50 scattering centers, which was less than that in the other targets. Meanwhile, M548 and M60 had numerous scattering centers. Thus, this imbalance in the number of scattering centers caused matching to be challenging, although valuable information was obtained for target identification.

For neighbor matching, the radius must be determined. Although the WVV descriptor is not affected by the translation of the scattering point, translation may affect the identification performance because only a part of the template remains based on the test point after neighbor matching. In Refs. [17,24], the researchers applied several different radii (e.g., 0.3, 0.4, and 0.5) for neighbor matching, and then the averages of all the similarities were employed to determine the final similarity between the test and its corresponding template.

In the experiment of this study, when the number of extracted scattering centers was small, the identification rate increased when a high value of 0.5 was used, rather than a low value of 0.3 m. Conversely, when a high value of 0.5 was used, there was no change in the identification rate, even when the number of scattering centers was large. We may need to consider the possibility of a translation transformation between the target and template before applying the neighbor-matching algorithm. Therefore, we used a single value of 0.5 so that the effect of the centering error of the test image could be mitigated.

**Figure 9:**
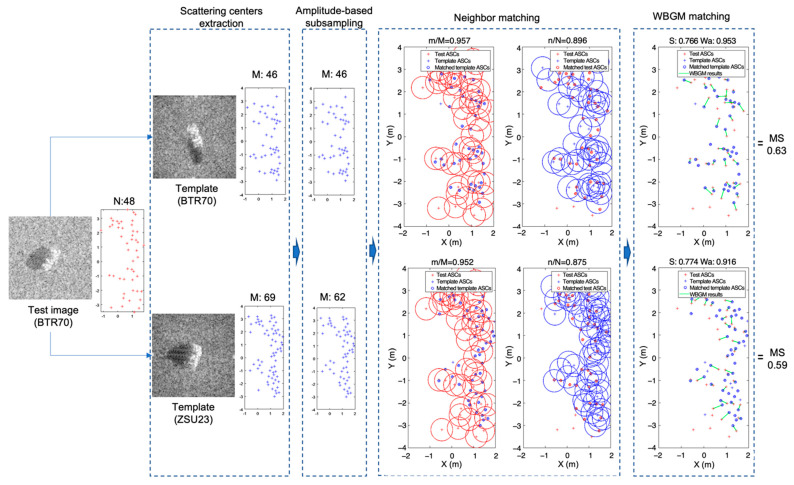
Example of recognition results obtained using the proposed algorithm.

Figure 9 shows an example of the processing according to the proposed algorithm (Figure 2), in which the test image BTR 70 was well recognized in the ambiguous template images (BTR 70 and ZSU23). First, the scattering points of each template image were selected in order of amplitude so that the number of scattering points of the test image did not exceed 48 × 1.3 (=62.4), where the parameter 1.3 means $A_{ratio}$, as mentioned in Section 2.2. Consequently, all 46 scattering points extracted from template BTR70 were selected, and only 62 of the 69 scattering points of template ZSU23 were selected. The point ratio selected by neighbor matching had a lower value for template ZSU23 than BTR70. The test ASCs and matched template ASCs were used to reconstruct the WVV set, and the similarity between the test and template WVV sets was measured. The WVV-based similarity ($S_{Norm}$) of template ZSU23 was higher than that of template BTR70. Contrastingly, the number of pairs matched by the WBGM of template ZSU23 was smaller than that of template BTR70; therefore, the $W_{a}$ of ZSU23 was lower. Finally, after WBGM, the matching score was calculated using Equation (9), giving a value of 0.63 for the template image of BTR70 and 0.59 for ZSU23. Although the WVV-based similarity alone did not show good recognition results, the performance was considerably improved using the proposed weights.

### 3.2. Standard Operating Condition

The proposed method was first evaluated under SOC on 10 classes of targets for overall classification accuracy. An actual image was used as the test target chip and a simulated image was used as the template. In the identification performance experiment using a synthetic image as a template, a simulated image can be used under the same observation conditions (depression angle) as the real image. However, owing to the characteristics of the SAR image, the distribution of scattering centers may vary depending on the imaging parameters, such as the depression angle or subtle changes in the pose of the target. In addition, because there is a difference between synthetic and real images, it is more advantageous for target identification to use not only the same observation angle data, but also adjacent observation angle data, as the template image. The merged training data were generated by combining the synthetic data with depression angles of 16° and 17° for 1052 chips.

Table 2 and Table 3 show the confusion matrix of the proposed method using a real 17° and 16° as the test image and a synthetic 16°/17° as the template. The performance was expressed by the percentage of correct classifications (PCCs). The average PCCs of all 10 targets in Table 2 were 90.7%, whereas the M2 target was recognized with PCCs under 80%, and the remaining targets were over 86%. For the result of real 16° data (Table 3), not only M2 but also BMP2 and M548 showed PCCs under 80%. Meanwhile, T72 and ZSU23 showed higher PCCs than the real 17° data. For real 16° and 17° data identifications, when only synthetic 16° and 17° data corresponding to each other were used as template data, the recognition rates were 87.0% and 88.0%, respectively. When synthetic 16°/17° data were used to identify real 16° and 17° data, the identification rates were improved to 87.3% and 90.7%, respectively. With the merged data from synthetic 16° and 17° angles, the real 17° data improved the recognition performance by 2.7%, while the real 16° data improved by only 0.3%.

**Figure 10:**
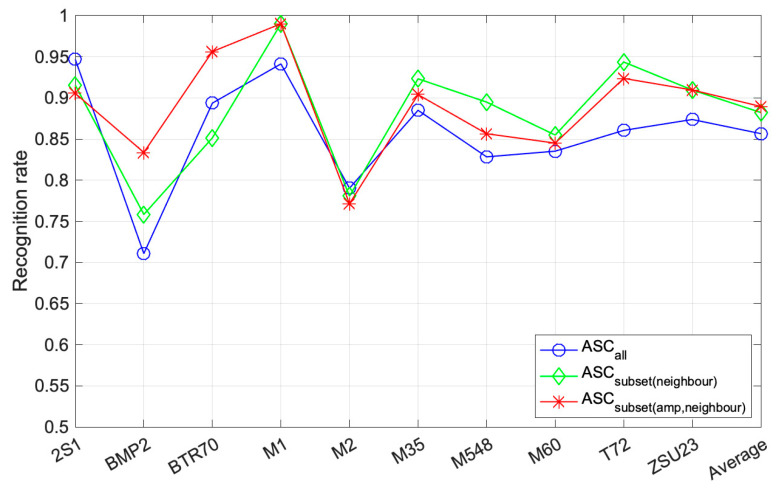
Recognition rate of each target according to different sets of scattering centers.

We attempted to prove the effectiveness of our proposed method using a subset of ASCs. There are three types of ASCs: $ASC_{all}$ means that all the test and template ASCs are used in WVV-based feature reconstruction as in Ref. [26], $ASC_{subset\left(neighbor\right)}$ indicates that the subset of ASCs is selected by neighbor matching, and $ASC_{subset\left(amp,neighbor\right)}$ indicates that the subset of ASCs is selected by amplitude-based sub-sampling followed by neighbor matching. Figure 10 shows the recognition rate of the 10 targets according to the ASC types used for classification. As described above, the merged data from synthetic 16° and 17° angles were used as a single template. This is the result of averaging the respective recognition performance obtained by using real 16° and 17° data as the test images. The overall performance of ${\mathrm{ASC}}_{\mathrm{all}}$ using all extracted ASCs suggested by Ref. [26] was lower than the results achieved by our proposed methods. When using the $ASC_{all}$, the recognition rate of BMP2 was very low, at about 70%. The $ASC_{subset\left(amp,neighbor\right)}$ showed a high recognition rate of about 85% or more in all targets except M2, and the performance variance for the 10 targets was the smallest among the three types of ASC. The results of $ASC_{subset\left(amp,neighbor\right)}$ were appropriate for the 10-class target classification, and in particular, the recognition rate of BMP2, BTR70, and T72 was greatly improved, by more than 5% compared to $ASC_{all}$.

### 3.3. Occlusion and Random Missed Pixels

**Figure 11:**
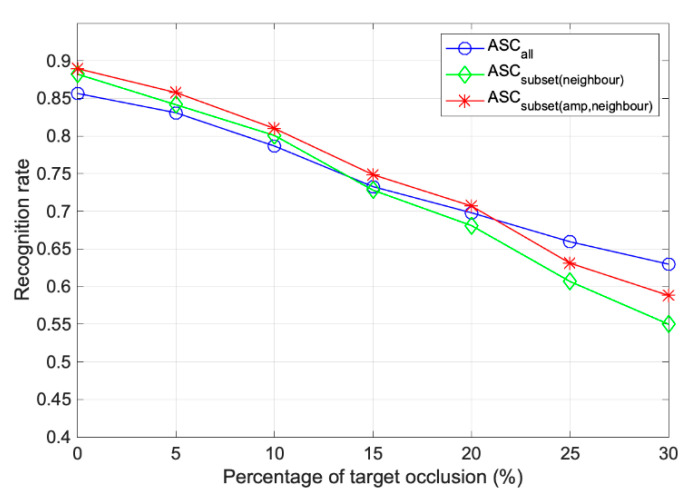
Performance comparison of different subsets by the degree of partial occlusion.

In the real world, the occlusion of a target by the external environment, such as artificial or natural objects, can always occur. The performance of the proposed method was investigated using directional occlusions. Similarly, the test samples in Table 1 were simulated to obtain samples with different levels of directional occlusion for classification. Figure 11 shows the recognition rate for each method with varying levels of directional occlusions. When the target was partially occluded, only part of the local structure was discriminative for the target. The WVV-based reconstruction with ASCs selected by neighbor matching made it possible to consider the local similarity between the test and the template.

When the test was occluded randomly from different directions, the performance of the proposed method, $ASC_{subset\left(amp,neighbor\right)}$, remained at a high level of over 70% until the occlusion level was 20%. Compared to the overall recognition rate of $ASC_{all}$, it was improved by 1–4%. When the WVV was reconstructed with a part selected as a neighbor rather than the entire scattering center, better results were obtained with less than 20% occlusion. In addition, the method applying $ASC_{subset\left(amp,neighbor\right)}$ was maintained at approximately 3% higher than the neighbor subset, although there was no considerable difference in value. However, when >25% occlusion occurred, it was better to use $ASC_{all}$. In the case of occlusion of 20% or more, the average recognition rate was lowered to 70% or less. That is, the overall reliability of the performance was too low to be used in practice. Therefore, it is better to use a subset instead of all ASCs, assuming a low occlusion rate, for a high recognition rate.

The ASCs could be interrupted by noise and differences in image resolution. A sensitivity analysis for randomly missed points was also performed. We randomly removed them according to the percentage of missed points. The remaining SCs were used to reconstruct WVV-based features that were matched with the templates. The percentage of missed points varied from 0 to 50%, and 10 Monte Carlo simulations were conducted for each percentage.

**Figure 12:**
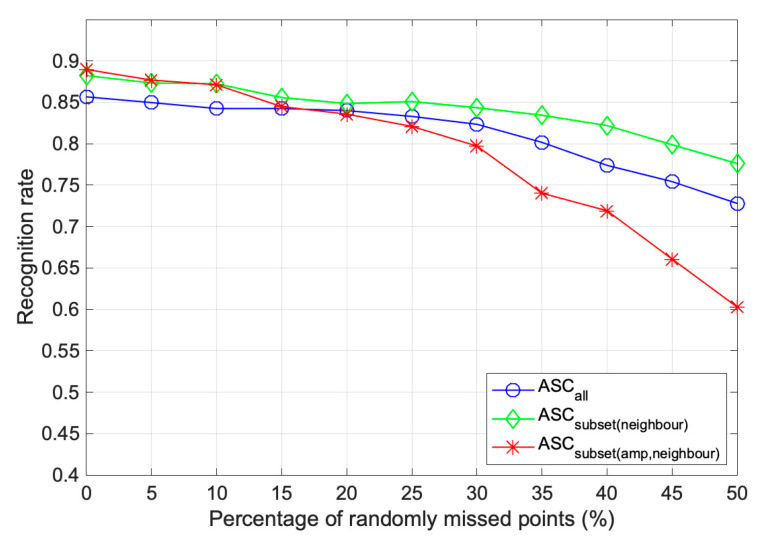
Performance comparison of the subsets by randomly missed points.

As shown in Figure 12, $ASC_{subset\left(neighbor\right)}$ always showed a higher recognition rate than $ASC_{all}$, verifying its robustness to random occlusion. The high performance of over 80% in $ASC_{subset\left(neighbor\right)}$ was maintained until a removal level of 45%. On the other hand, $ASC_{subset\left(amp,neighbor\right)}$ showed better results than $ASC_{all}$ only up to 15% random occlusion. This is because the ASCs in the test image were randomly removed, while the ASCs in the template image were removed based on the amplitude of ASCs, so the relevance between the remaining pixels decreased significantly as the percentage of random occlusion increased.

We achieved high performances using the SAMPLE dataset, with 100% synthetic data in the training set. When the experiments were conducted by changing the types of ASCs used, the overall recognition rate under SOC (no occlusion and no random missed pixels) was improved about 4% compared to $ASC_{all}$. Additionally, the proposed method was less sensitive to a small amount of partial occlusion and random pixel removal.

We compared the proposed method with existing methods [39] to illustrate its performance. The experimental results, where the average recognition rate was 24.97% when solely synthetic data were used in the training batch, were initially presented by Ref. [31], the creators of the SAMPLE dataset. Their algorithm was based on a convolutional neural network (CNN). They then achieved average accuracies of 51.58% by training DenseNet with the assistance of a generative adversarial network (GAN) in their most recent work [25]. Their deep learning-based performances seem to be superior to our proposed method in Refs. [51,52]. However, the accuracy dropped notably below 85% when they used 100% synthetic data in training. Consequently, our proposed method had a higher performance, which was also more stable when using 100% synthetic data as the training dataset.

In terms of the validity under EOCs, our performances were also compared to other previous works where they used the SAMPLE dataset for experiments, but the organization of data setting (number of classes, depression angle, etc.) was not same as our dataset. For example, the target recognition rate decreased by 30% during 50% random occlusion in Ref. [17], when they use a measured dataset (MSTAR) for both testing and training, but only by 10.6% in our study using $ASC_{subset\left(neighbor\right)}$. This was the best performance under random occlusion among the previous works. Compared to the results of other methods, our similarity measure can effectively overcome the recognition difficulties caused by random occlusion when $ASC_{subset\left(neighbor\right)}$ is used, though the same condition with our dataset was not applied in the methods. We believe that the advantage of the proposed algorithm is that it mostly focuses on the local features related to the intersected target parts to improve the feasibility of the SAR ATR system in response to a realistic scenario. However, there are several limitations resulting from human intervention. One is the existence of an empirical threshold for amplitude-based subsampling, and the need to set a specific radius in neighbor matching, which can cause significant performance degradation in our algorithm when most parts of the target are occluded. To resolve these limitations, we will also consider how to integrate global similarities with the proposed method in the case of target occlusion.

## 4. Conclusions

A robust algorithm is required to identify partial differences between real and synthetic images to perform target identification based on a dataset of synthetic images, such as SAMPLE. We proposed an improved WVV-based ATR method using a subset of the template’s ASCs, using the ASCs’ amplitude and the proximity of the scattering center location, which is less susceptible to the partial differences between two ASC sets. The SAMPLE dataset, with 10 classes of military targets, was used in the experiments. With the merged template of synthetic 16°/17° images, the recognition rates were 87.3% for the 16° real images and 90.7% for the 17° real images. The performance with occlusion remained above 70% until the occlusion level reached 20%. In addition, the subset of ASCs selected by neighbor matching achieved recognition rates great than 80% until the proportion of randomly missed points reached 45%. Therefore, we expect that the proposed method will be useful in practical SAR ATR systems using synthetic images with partial and random differences in ASCs due to occlusion.

## Figures and Tables

**Table 1:** Number of SAR images in the test and training sets of the SAMPLE dataset.

Type	2S1	BMP2	BTR70	M1	M2	M35	M60	M548	T72	ZSU23
Test set (real 16°) Train set (syn 16°)	50	55	43	52	52	52	52	51	56	50
Test set (real 17°) Train set (syn 17°)	58	52	49	51	53	53	53	60	52	58

**Table 2:** Recognition results obtained using the proposed method within 10 target classification tests (test image: real 17°, template image: synthetic 16°/17°).

Type	2S1	BMP2	BTR70	M1	M2	M35	M548	M60	T72	ZSU23	PCC (%)
2S1	54	0	1	0	1	0	0	0	0	2	93.10
BMP2	4	47	1	0	0	0	0	0	0	0	90.38
BTR70	2	0	47	0	0	0	0	0	0	0	95.92
M1	0	0	0	50	1	0	0	0	0	0	98.04
M2	4	4	0	0	41	0	0	0	1	3	77.36
M35	0	0	0	0	0	52	1	0	0	0	98.11
M548	0	0	0	0	0	3	49	0	0	1	92.45
M60	3	0	0	4	0	0	0	52	1	0	86.67
T72	1	3	0	0	1	0	0	2	45	0	86.54
ZSU23	6	0	0	0	1	0	0	0	0	51	87.93
Average											90.65

**Table 3:** Recognition results obtained using the proposed method within 10 target classification tests (test image: real 16°; template image: synthetic 16°/17°).

Type	2S1	BMP2	BTR70	M1	M2	M35	M548	M60	T72	ZSU23	PCC (%)
2S1	44	0	2	0	0	0	0	0	0	4	88.00
BMP2	10	42	1	0	2	0	0	0	0	0	76.36
BTR70	0	0	41	0	1	0	0	0	0	1	95.35
M1	0	0	0	52	0	0	0	0	0	0	100.00
M2	4	3	0	0	40	0	0	0	1	4	76.92
M35	0	0	7	0	0	43	0	0	0	2	82.69
M548	0	0	7	0	0	2	41	0	0	2	78.85
M60	3	0	0	5	0	0	0	42	1	0	82.35
T72	0	0	0	0	0	0	0	1	55	0	98.21
ZSU23	2	0	1	0	0	0	0	0	0	47	94.00
Average											87.27

## Data Availability

The SAMPLE dataset was obtained in accordance with the instructions contained in Ref. [31] and is available online: https://github.com/benjaminlewis-afrl/SAMPLE_dataset_public (accessed on 25 August 2022).

## References

[1] Wang L., Bai X., Zhou F. (2019). SAR ATR of ground vehicles based on ESENet. Remote Sens..

[2] Brusch S., Lehner S., Fritz T., Soccorsi M., Soloviev A., van Schie B. (2010). Ship surveillance with TerraSAR-X. IEEE Trans. Geosci. Remote Sens..

[3] Song D., Zhen Z., Wang B., Li X., Gao L., Wang N., Xie T., Zhang T. (2020). A novel marine oil spillage identification scheme based on convolution neural network feature extraction from fully polarimetric SAR imagery. IEEE Access.

[4] Fu K., Dou F.-Z., Li H.-C., Diao W.-H., Sun X., Xu G.-L. (2018). Aircraft recognition in SAR images based on scattering structure feature and template matching. IEEE J. Sel. Top. Appl. Earth Obs. Remote Sens..

[5] El-Darymli K., Gill E.W., Mcguire P., Power D., Moloney C. (2016). Automatic target recognition in synthetic aperture radar imagery: A state-of-the-art review. IEEE Access.

[6] Moreira A., Prats-Iraola P., Younis M., Krieger G., Hajnsek I., Papathanassiou K.P. (2013). A tutorial on synthetic aperture radar. IEEE Geosci. Remote Sens. Mag..

[7] Park J.-H., Seo S.-M., Yoo J.-H. (2021). SAR ATR for limited training data using DS-AE network. Sensors.

[8] Paulson C., Nolan A., Goley S., Nehrbass S., Zelnio E. (2019). Articulation study for SAR ATR baseline algorithm. Algorithms for Synthetic Aperture Radar Imagery XXVI.

[9] Kechagias-Stamatis O., Aouf N. (2021). Automatic target recognition on synthetic aperture radar imagery: A survey. IEEE Aerosp. Electron. Syst. Mag..

[10] Dogaru T., Phelan B., Liao D. (2019). Imaging of buried targets using UAV-based, ground penetrating, synthetic aperture radar. Radar Sensor Technology XXIII.

[11] Ross T.D., Bradley J.J., Hudson L.J., O’connor M.P. (1999). SAR ATR: So what’s the problem? An MSTAR perspective. Algorithms for Synthetic Aperture Radar Imagery VI.

[12] Keydel E.R., Lee S.W., Moore J.T. (1996). MSTAR extended operating conditions: A tutorial. Algorithms Synth. Aperture Radar Imag. III.

[13] Chen S., Wang H., Xu F., Jin Y.-Q. (2016). Target classification using the deep convolutional networks for SAR images. IEEE Trans. Geosci. Remote Sens..

[14] Dudgeon D.E., Lacoss R.T. (1993). An Overview of Automatic Target Recognition. Linc. Lab. J..

[15] Liang W., Zhang T., Diao W., Sun X., Zhao L., Fu K., Wu Y. (2020). SAR target classification based on sample spectral regularization. Remote Sens..

[16] Feng S., Ji K., Zhang L., Ma X., Kuang G. (2021). SAR target classification based on integration of ASC parts model and deep learning algorithm. IEEE J. Sel. Top. Appl. Earth Obs. Remote Sens..

[17] Lu C., Fu X., Lu Y. (2021). Recognition of occluded targets in SAR images based on matching of attributed scattering centers. Remote Sens. Lett..

[18] Potter L.C., Moses R.L. (1997). Attributed scattering centers for SAR ATR. IEEE Trans. Image Process..

[19] Fan J., Tomas A. (2018). Target Reconstruction Based on Attributed Scattering Centers with Application to Robust SAR ATR. Remote Sens..

[20] Liu Z., Wang L., Wen Z., Li K., Pan Q. (2022). Multi-Level Scattering Center and Deep Feature Fusion Learning Framework for SAR Target Recognition. IEEE Trans. Geosci. Remote Sens..

[21] Ding B., Wen G., Ma C., Yang X. (2018). An efficient and robust framework for SAR target recognition by hierarchically fusing global and local features. IEEE Trans. Image Process..

[22] Lv J., Liu Y. (2019). Data augmentation based on attributed scattering centers to train robust CNN for SAR ATR. IEEE Access.

[23] Feng S., Ji K., Wang F., Zhang L., Ma X., Kuang G. (2022). Electromagnetic Scattering Feature (ESF) Module Embedded Network Based on ASC Model for Robust and Interpretable SAR ATR. IEEE Trans. Geosci. Remote Sens..

[24] Ding B., Wen G., Zhong J., Ma C., Yang X. (2016). Robust method for the matching of attributed scattering centers with application to synthetic aperture radar automatic target recognition. J. Appl. Remote Sens..

[25] Lewis B., DeGuchy O., Sebastian J., Kaminski J. (2019). Realistic SAR data augmentation using machine learning techniques. Algorithms for Synthetic Aperture Radar Imagery XXVI.

[26] Tian S., Yin K., Wang C., Zhang H. (2015). An SAR ATR method based on scattering centre feature and bipartite graph matching. IETE Tech. Rev..

[27] Ding B., Wen G. (2018). Target reconstruction based on 3-D scattering center model for robust SAR ATR. IEEE Trans. Geosci. Remote Sens..

[28] Diemunsch J.R., Wissinger J. (1998). Moving and stationary target acquisition and recognition (MSTAR) model-based automatic target recognition: Search technology for a robust ATR. Algorithms for synthetic aperture radar Imagery V.

[29] Camus B., Barbu C.L., Monteux E. (2022). Robust SAR ATR on MSTAR with Deep Learning Models trained on Full Synthetic MOCEM data. arXiv.

[30] Vernetti A., Scarnati T., Mulligan M., Paulson C., Vela R. Target Pose Estimation using Fused Radio Frequency Data within Ensembled Neural Networks. Proceedings of the 2022 IEEE Radar Conference (RadarConf22).

[31] Lewis B., Scarnati T., Sudkamp E., Nehrbass J., Rosencrantz S., Zelnio E. (2019). A SAR dataset for ATR development: The Synthetic and Measured Paired Labeled Experiment (SAMPLE). Algorithms for Synthetic Aperture Radar Imagery XXVI.

[32] Arnold J.M., Moore L.J., Zelnio E.G. (2018). Blending synthetic and measured data using transfer learning for synthetic aperture radar (SAR) target classification. Algorithms for Synthetic Aperture Radar Imagery XXV.

[33] Inkawhich N., Inkawhich M.J., Davis E.K., Majumder U.K., Tripp E., Capraro C., Chen Y. (2021). Bridging a gap in SAR-ATR: Training on fully synthetic and testing on measured data. IEEE J. Sel. Top. Appl. Earth Obs. Remote Sens..

[34] Bhanu B., Jones G. Target recognition for articulated and occluded objects in synthetic aperture radar imagery. Proceedings of the Proceedings of the 1998 IEEE Radar Conference, RADARCON’98. Challenges in Radar Systems and Solutions (Cat. No.98CH36197).

[35] Murtagh F. (1992). A new approach to point pattern matching. Publ. Astron. Soc. Pac..

[36] Chen Y., Huang D., Xu S., Liu J., Liu Y. (2022). Guide Local Feature Matching by Overlap Estimation. arXiv.

[37] Sarlin P.-E., DeTone D., Malisiewicz T., Rabinovich A. Superglue: Learning feature matching with graph neural networks. Proceedings of the IEEE/CVF Conference on Computer Vision and Pattern Recognition.

[38] Yun D.-J., Lee J.-I., Bae K.-U., Song W.-Y., Myung N.-H. (2018). Accurate Three-Dimensional Scattering Center Extraction for ISAR Image Using the Matched Filter-Based CLEAN Algorithm. IEICE Trans. Commun..

[39] Araujo G.F., Machado R., Pettersson M.I. (2022). Non-Cooperative SAR Automatic Target Recognition Based on Scattering Centers Models. Sensors.

[40] Högbom J. (1974). Aperture synthesis with a non-regular distribution of interferometer baselines. Astron. Astrophys. Suppl. Ser..

[41] Ding B., Wen G. (2019). Combination of global and local filters for robust SAR target recognition under various extended operating conditions. Inf. Sci..

[42] Ding B., Wen G., Zhong J., Ma C., Yang X. (2017). A robust similarity measure for attributed scattering center sets with application to SAR ATR. Neurocomputing.

[43] Tang T., Su Y. Object recognition based on feature matching of scattering centers in SAR imagery. Proceedings of the 2012 5th International Congress on Image and Signal Processing.

[44] Ozdemir C. (2012). Inverse Synthetic Aperture Radar Imaging with MATLAB Algorithms.

[45] Demirkaya O., Asyali M.H., Sahoo P.K. (2008). Image Processing with MATLAB: Applications in Medicine and Biology.

[46] Novak L.M., Owirka G.J., Brower W.S. (2000). Performance of 10-and 20-target MSE classifiers. IEEE Trans. Aerosp. Electron. Syst..

[47] Karine A., Toumi A., Khenchaf A., El Hassouni M. (2018). Radar target recognition using salient keypoint descriptors and multitask sparse representation. Remote Sens..

[48] Yu J., Zhou G., Zhou S., Yin J. (2021). A Lightweight Fully Convolutional Neural Network for SAR Automatic Target Recognition. Remote Sens..

[49] Gao F., Huang T., Sun J., Wang J., Hussain A., Yang E. (2019). A new algorithm for SAR image target recognition based on an improved deep convolutional neural network. Cogn. Comput..

[50] Choi Y. (2022). Simulated SAR Target Recognition using Image-to-Image Translation Based on Complex-Valued CycleGAN. J. Korean Inst. Inf. Technol..

[51] Inkawhich N.A., Davis E.K., Inkawhich M.J., Majumder U.K., Chen Y. (2021). Training SAR-ATR models for reliable operation in open-world environments. IEEE J. Sel. Top. Appl. Earth Obs. Remote Sens..

[52] Sellers S.R., Collins P.J., Jackson J.A. Augmenting simulations for SAR ATR neural network training. Proceedings of the 2020 IEEE International Radar Conference (RADAR).

